# A parameter-optimization framework for neural decoding systems

**DOI:** 10.3389/fninf.2023.938689

**Published:** 2023-02-02

**Authors:** Jing Xie, Rong Chen, Shuvra S. Bhattacharyya

**Affiliations:** ^1^Department of Electrical and Computer Engineering, University of Maryland at College Park, College Park, MD, United States; ^2^Department of Diagnostic Radiology and Nuclear Medicine, University of Maryland at Baltimore, Baltimore, MD, United States; ^3^Institute for Advanced Computer Studies (UMIACS), University of Maryland at College Park, College Park, MD, United States

**Keywords:** parameter optimization, neural decoding, real-time image processing, dataflow, data stream mining

## Abstract

Real-time neuron detection and neural activity extraction are critical components of real-time neural decoding. They are modeled effectively in dataflow graphs. However, these graphs and the components within them in general have many parameters, including hyper-parameters associated with machine learning sub-systems. The dataflow graph parameters induce a complex design space, where alternative configurations (design points) provide different trade-offs involving key operational metrics including accuracy and time-efficiency. In this paper, we propose a novel optimization framework that automatically configures the parameters in different neural decoders. The proposed optimization framework is evaluated in depth through two case studies. Significant performance improvement in terms of accuracy and efficiency is observed in both case studies compared to the manual parameter optimization that was associated with the published results of those case studies. Additionally, we investigate the application of efficient multi-threading strategies to speed-up the running time of our parameter optimization framework. Our proposed optimization framework enables efficient and effective estimation of parameters, which leads to more powerful neural decoding capabilities and allows researchers to experiment more easily with alternative decoding models.

## 1. Introduction

Neural decoding based on neuroimaging signals is an important tool for understanding neural codes for studying and treating brain disorders, such as Alzheimer's disease and Parkinson's disease. Neural decoding systems in general have many parameters, including hyperparameters associated with machine learning sub-systems. The parameters induce a complex design space, where alternative configurations (design points) provide different trade-offs involving key operational metrics, including accuracy and time-efficiency. Parameter optimization of neural decoding systems is important for achieving strategic trade-offs between accuracy and time-efficiency. For example, for off-line neural signal analysis, it is typically desirable to optimize parameters to favor high accuracy at the expense of relatively long running time. On the other hand, for real-time analysis, parameter optimization would typically be geared towards maximizing accuracy subject to strict execution time constraints. Real-time neural decoding is useful, for example, in precision neuromodulation systems, where stimulation to the brain must delivered in a timely manner in relation to the current state of brain activity. The diverse trade-offs that must be considered in neural decoding — depending on the application scenario — creates a need for flexible and effective parameter optimization that can be used to efficiently navigate the design spaces for configuring neural decoding systems.

Neural decoding systems may involve both continuous-valued parameters as well as discrete-valued parameters. In this work, we refer to *parameter optimization* as the optimization of parameter values for parameter sets that are in general hybrid combinations of continuous and discrete parameters. Our view of parameter optimization is therefore more general than parameter “tuning,” which is typically associated only with continuous-valued parameters.

Parameter optimization for neural decoding is challenging due to the complexity of the underlying design spaces, as described above. With manual approaches, which are conventionally used, system designers may be effective in selecting very high level parameters, such as the types of decoding or preprocessing algorithms to be used; however, it is extremely time consuming to study a wide range of alternative design points in a way that comprehensively takes into account the impact of and interactions between diverse sets of relevant parameters. Moreover, conventional parameter optimization approaches typically consider only algorithmic parameters, whereas dataflow parameters, which have significant impact of time-efficiency, are not considered. Here, by *dataflrow* parameters, we mean parameters associated with executing the different signal processing modules in a neural decoding system on the targeted hardware platform.

In this paper, we develop an optimization framework for automated and holistic parameter optimization of neural decoding systems. The framework considers both algorithmic and dataflow parameters while jointly taking into account both the neural decoding accuracy and execution time of the optimized solutions. The proposed framework applies a population-based search strategy to optimize the relevant algorithmic and dataflow parameters of the given neural decoding system. The framework is general in that a variety of search strategies can be plugged-in; it is not specific to a single type of search method. To demonstrate this generality, we apply two different search strategies in our experiments — Particle Swarm Optimization (PSO), which is a randomized search strategy that is effective for navigating nonlinear design spaces that are based on diverse types of parameters (Kennedy and Eberhart, 1995), and Genetic Algorithms (GAs), which is a metaheuristic method that uses different kinds of biologically inspired operators, such as mutation, crossover and selection, for evolving successive generations of populations (sets of candidate solutions). We present a prototype implementation of the proposed framework in a novel software tool, which we refer to as the NEural DEcoding COnfiguration (NEDECO) package, since the objective of the package is to help experimental neuroscientists and neural decoding system designers to arrive at strategically-optimized configurations of neural decoding implementations.

NEDECO operates by iteratively executing alternative neural decoding configurations to assess their performance and feed back the assessment to help derive new candidate configurations to evaluate. This approach of feedback-driven design space exploration is carried out based on the PSO and GA methodology. To accelerate the evaluation of neural decoding configurations, we exploit their dataflow models to derive efficient multi-threaded executions of the configurations on commodity, off-the-shelf desktop or laptop computers that employ multicore processors. We perform an extensive experimental evaluation of NEDECO in which we compare its parameter optimization results to the manually-optimized parameter configurations for two previously published systems for neural decoding. Our results demonstrate the effectiveness of NEDECO in deriving neural decoding implementations that offer significantly improved trade-offs between decoding accuracy and the speed at which decoding is performed.

The remainder of this paper is organized as follows. Section 2 presents related work in neural decoding and summarizes the contribution of this paper in the context of the related work. In Section 3, we introduce the architecture of NEDECO, including the models and methods for automated parameter optimization and the methods for accelerating the parameter optimization process through efficient use of parallel processing resources. In Section 4, we introduce the experimental methodology that we use to demonstrate and evaluate NEDECO, and we present the results of our experimental evaluation, which concretely demonstrate the effectiveness of NEDECO when it is applied for parameter optimization to multiple state-of-the-art neural decoding tools. Finally, in Section 5, we summarize the developments of the paper, and discuss limitations of NEDECO and directions for future work.

## 2. Background and related work

A number of previous research efforts have studied parameter tuning for neural decoding. For example, Pnevmatikakis et al. proposed a method for automatically tuning a selected subset of parameters, including the calcium indicator dynamics and time-varying baseline concentration, but the remaining parameters still require manual tuning (Pnevmatikakis et al., 2016). Moreover, there is no systematic integration between the human-tuned parameters and the optimization of parameter values for the parameter-subset that is automatically tuned. Giovannucci et al. eliminate the hyper-parameter tuning in the a convolutional neural network (CNN) classifier subsystem within their proposed neural decoding model (Giovannucci et al., 2019). However, like the work of Pnevmatikakis et al., the tuning process developed by Giovannucci et al. targets a subset of parameters and requires a separate human-driven tuning step for the remaining parameters. Additionally, and perhaps most significantly, the parameter tuning processes in Pnevmatikakis et al. (2016) and Giovannucci et al. (2019) are based on the image analysis models and algorithms that they apply for their neural decoding systems. The automation strategy cannot be directly applied to other analysis models for neural decoding.

Glaser et al. (2020) present a tutorial and accompanying software package to assist neuroscientists in applying machine learning methods to neural decoding problems. While the machine learning methods integrated in the software package outperforms conventional neural decoding methods, the work of Glaser et al. (2020) does not address the problem of automated parameter tuning.

Liu et al. (2011) proposed a real-time PSO method for power system optimization. In this method, a novel approach was developed to accelerate the particle evaluation (fitness evaluation) process. The approach of Liu is representative of various works that focus on fitness function acceleration. In contrast, NEDECO is designed as a higher-level framework that can be integrated with a variety of search strategies and fitness functions, and is developed and demonstrated based on specific constraints and requirements that are involved in the design and implementation of real-time neural decoding systems.

PSO methods have also been investigated for hyper-parameter optimization in deep neural networks (Lorenzo et al., 2017; Yamasaki et al., 2017; Guo et al., 2020; Li and Zhang, 2020; Singh et al., 2021). In contrast to these methods, our proposed NEDECO framework can be applied to neural decoding systems that use any type of machine learning model (not limited to DNN models); NEDECO can apply other search strategies (not just PSO); and our work is the first to apply such a general and comprehensive parameter optimization framework to neural decoding, which is a useful contribution to computational neuroscience.

To the best of our knowledge, NEDECO is the first software tool for generalized, automated tuning of parameters in calcium-imaging-based neural decoding systems. By “generalized”, we mean that the tool works comprehensively over all decoding algorithm parameters, works across a wide variety of model types and associated information extraction algorithms rather than being restricted to a specific type of model, and takes into account both the neural decoding accuracy and run-time efficiency. In Section 4, we demonstrate the flexibility of NEDECO by applying it to two significantly different neural decoding tools from the literature — the Neuron Detection and Signal Extraction Platform (NDSEP) (Lee et al., 2020), and CellSort (Mukamel et al., 2009), using two different methods as the underlying search strategy — particle swarm optimization and genetic algorithms. Our experiments demonstrate the ability of NEDECO to derive parameter settings that lead to significantly improved neural decoding performance compared to the previous published results using NDSEP and CellSort, which are based on hand-tuned parameters.

## 3. Proposed method

NEDECO applies PSO for optimizing heterogeneous collections of neural decoding system parameters, including continuous and discrete parameters. Additionally, the PSO techniques of NEDECO are implemented within a dataflow framework. This facilitates the retargetability of the framework to different neural decoding algorithms, and different platforms for optimization, and also facilitates the acceleration of the optimization process on multi-core computing platforms. The latter feature — acceleration of the optimization process — is important because the the process is computationally intensive, and higher quality solutions can generally be produced within a given time period if more efficient execution of the optimization engine is enabled. In Section 3.1, Section 3.2, and Section 3.4, we review fundamentals of PSO, GAs, and dataflow modeling, which are key foundations of NEDECO.

### 3.1. Particle swarm optimization

PSO is a form of population-based, randomized, iterative computation for optimization in the context of complex, multidimensional search spaces in which different dimensions of a search space may have very different characteristics and underlying data types (Kennedy and Eberhart, 1995). Inspired by the social behavior of animal flocks, populations in PSOs are referred to as *swarms*, and each member of a population (swarm) is referred to as a *particle*. Each particle represents a candidate solution in the context of the optimization problem that the enclosing PSO is designed to solve. Operation of a PSO involves tracking the positions of particles in the current swarm, and iteratively generating a new swarm from the previous one, which leads to successive generations of swarms. Intuitively, as swarms evolve, the quality of solutions represented by the candidate solutions will tend to increase, which leads to capability of the method to derive optimized solutions.

In general, the position of a particle in a PSO is an encoding of the candidate solution. In NEDECO, particles move in an *n*-dimensional space, where *n* is the number of parameters to jointly optimize in the given neural decoding system. Each dimension corresponds to a distinct parameter. The component of the position vector in a given dimension gives the value of the corresponding parameter. This can be viewed as a direct way of mapping candidate solutions for a parameter optimization problem into particles (position vectors) for a PSO.

Particles move (transition from one position to another) based on velocity vectors that are maintained along with the particles. The velocities are typically influenced by neighboring particles as well as by a current “best” particle within the population. A best particle is simply one whose associated design point maximizes the function that the PSO is designed to optimize among all elements of the current population. Here, we are assuming that the optimization objective is one of maximization; the approach described here can be adapted easily for minimization contexts.

Multiple particles may be tied to achieve the maximum function value. In the case of such a tie, one of the maximizing particles may be randomly selected as the best for the purpose of evolving the current PSO population (that is, for exerting influence on other particles' velocities). In NEDECO, this is the process by which multiple best particles are handled; although in general, there are other ways of handling ties.

As described in Section 3.1, the velocity of a particle *p* is used to update its position, and the velocity is determined both by neighboring particles of *p* in the swarm, as well as by the current best particle in the population. The concept of a neighboring particle is determined by the *topology*, which is a parameter of the PSO. The topology can be represented by a graph. In the *connectivity graph*, the vertices are in one-to-one correspondence with the particles, and two vertices are connected by an edge if the associated particles are neighbors in the given topology. For example, in a fully connected topology, all particles other than *p* are considered neighbors of *p*, while for a ring topology, the connectivity graph is ring-structured, and therefore, each particle has exactly two neighbors. A third commonly-used topology is the random topology, as defined by Clerc (2007). A random topology involves a dynamically evolving connectivity graph, which is initialized with randomly-placed connections (edges). The connectivity graph for the random topology is modified (again using randomization techniques) whenever there is no improvement in the population's best particle after a given transition from one generation of particles to the next. Such a transition is known as an *iteration* of the PSO. Use of a random or ring topology can help a PSO to further avoid getting stuck in local optima.

### 3.2. Genetic Algorithms

Like a PSO particle, a candidate solution is an “individual” in a population within a GA. Individuals in GAs are also referred to as “chromosomes.” When a GA is used the search strategy in NEDECO, each GA population contains a set of chromosomes, where each chromosome encapsulates a configuration of PNDS parameters. Initially, populations are randomly generated within the search space. To iteratively evolve toward better solutions, chromosomes within populations undergo random alterations (mutations). Additionally, pairs of chromosomes are randomly selected as “parents” from which new individuals are derived; this processes is referred to in GA terminology as “crossover.” In each GA iteration (population generation) the PNDS is used to evaluate each individual of the current population. The fitness is used to determine the probability that a given individual is selected as a parent for crossover — that is, whether or not the individual will influence the next generation of the GA execution. For more details on GAs, we refer the reader to Back et al. (1997).

In our GA implementation for use with NEDECO, six selection methods are supported. These include proportional roulette wheel selection (RWS), stochastic universal sampling (SUS), classic linear rank-based selection (RNK), linear rank-based selection with selective pressure (RSP), tournament selection (TNT), and transform ranking selection (TRS). Additionally, three crossover methods are supported, including one-point crossover (P1XO), two-point crossover (P2XO), uniform cross-over (UXO), and three mutation methods are supported, including boundary mutation (BDM), single-point mutation (SPM), and uniform mutation (UNM).

### 3.3. Multiobjective optimization

NEDECO is designed to take into account two metrics for optimization of parameters in a given neural decoding system — both the accuracy of neural detection and the run-time efficiency of the process. In terms of calcium imaging, this joint consideration of both accuracy and efficiency is one of the key innovative aspects of NEDECO. NEDECO applies a linear aggregation approach (Parsopoulos and Vrahatis, 2002) to weighting the objectives so that the user (neural decoding system designer) can adjust the relative importance levels given to the two metrics based on application requirements. For example, a neural decoding system that is deployed in a closed-loop neuromodulation system would typically have an increased weighting given to run-time efficiency, whereas an offline system for batch processing of neuroscience datasets may have much higher relative weighting for accuracy. The framework also gives the system designer a simple means to tune the final achieved trade-off. In particular, the NEDECO optimization process can be iteratively re-executed — with the relative weightings varied between iterations — in case the system designer would like to evaluate different implementation options in terms of the provided trade-offs.

We apply a linear aggregation approach to handling multiple objectives in NEDECO because a linear approach is intuitively easy for the system designer to understand, especially when a small number of different objective metrics is involved. A general issue that may be considered when extending PSOs and GAs to multiobjective design optimization contexts is that of extending the concept of best solutions to encompass solution subsets that are non-dominated in the sense of Pareto optimization (Zhou et al., 2011). However, this issue is avoided when *aggregating functions* are applied to map metric values in multiple dimensions into single-dimensional values (e.g., into real numbers). Among aggregating approaches, linear aggregation is perhaps easiest to understand and most commonly used. This approach defines the aggregating function as a linear combination *a*_1_*m*_1_+*a*_2_*m*_2_ + … + *a*_*q*_*m*_*q*_ of the (possibly normalized) metric values *m*_1_, *m*_2_, …, *m*_*q*_ for a given candidate solution, where there are *q* dimensions in the design evaluation space. Here *a*_1_, *a*_2_, …, *a*_*q*_ are coefficients of the linear aggregation; in NEDECO, *q* = 2, and the coefficients are defined as *a*_1_ = *z* and *a*_2_ = (1−*z*), where *z* is a single parameter that the system designer uses to control the relative weighting given to the two metrics, accuracy and run-time efficiency.

Various alternative approaches have been developed to handle multiobjective optimization in PSOs and GAs, such as the hyper-volume indicator approaches, which are standard in multiobjective analysis using Pareto fronts. These approaches are more sophisticated compared to linear aggregation [e.g., see Miettinen (1999), Jin et al. (2001), Clerc and Kennedy (2002), Parsopoulos and Vrahatis (2002), Dimanov et al. (2021)]. Investigation of such approaches in the context of NEDECO is an interesting direction for future work.

### 3.4. Dataflow modeling

NEDECO is designed based on dataflow modeling concepts — particularly, on a form of dataflow modeling for signal and information processing systems that is called core functional dataflow (Plishker et al., 2009). Dataflow is a model of computation that is widely used in the design of signal and information processing systems, including, in recent years, in the design of systems for neural decoding (Lee et al., 2020).

In dataflow-based application modeling, applications are represented as directed graphs in which the vertices, called *actors* represent functional modules and each edge represents first-in, first-out (FIFO) communication of data from one actor to another (Lee and Parks, 1995). Each unit of data that is communicated along a dataflow edge is referred to as a *token*. These tokens can have arbitrary data types associated with them, such as integers, floating point numbers, pointer types, and arbitrary classes in object-oriented programming languages.

Actors are executed in terms of discrete units of execution, which are referred to as *firings*, where the discrete units are typically defined in terms of the numbers of tokens that are produced and consumed from the edges. For example in synchronous dataflow (SDF), which is an important specialized form of dataflow, each actor produces and consumes a constant number of actors on each incident edge (Lee and Messerschmitt, 1987). The numbers of tokens produced and consumed by an SDF actor can vary from one incident edge to another (and among different actors), but for each incident edge, the number must be constant for all firings of the actor.

Dataflow representations are useful for formally representing the high-level signal flow and computational organization of signal and information processing systems. A wide variety of methods have been developed for analysis and design optimization of system implementations that are derived using dataflow representations (Bhattacharyya et al., 2019). Due to the precise manner in which components are interfaced — based on connectivity in the dataflow graph and characterizations of token production and consumption — dataflow-based representations also facilitate “plug-and-play” signal processing architectures, where different versions of an actor subsystem can be used at different times during execution or in different versions of the system. This enhanced modularity is exploited in NEDECO to make the tool easy to retarget for parameter optimization of different neural decoding systems.

For more background on dataflow modeling, analysis and design optimization for signal and information processing systems, we refer the reader to Lee and Parks (1995) and Bhattacharyya et al. (2019), and for details on how dataflow methods can be applied to efficient neural decoding, we refer the reader to Lee et al. (2020).

### 3.5. Core functional dataflow

As mentioned in Section 3.4, NEDECO applies a form of dataflow called core functional dataflow (CFDF), which is useful for designing and integrating dataflow actors that involve different modes of operation. A CFDF actor *A* has an associated set of modes *M*(*A*) = {μ_1_, μ_2_, …, μ_*c*(*A*)_}, where *c*(*A*) denotes the number of modes in *A*. Each mode μ_*i*_ corresponds to a distinct computational function that is to be performed by the actor. Each firing *f* of a CFDF actor *A* has associated with it a unique mode κ(*f*) ∈ *M*(*A*), which governs the type of computation that is to be performed during the firing.

For each dataflow edge *e* that is incident to *A*, the dataflow rate associated with *A* and *e* is constant for a given mode. Here, by the *dataflow rate*, we mean the number of tokens consumed by *A* from *e* if *e* is an input edge of *A*, and the number of tokens produced by *A* onto *e* if *e* is an output edge. CFDF is therefore similar to SDF in its requirement of constant dataflow rates. However, CFDF is more flexible in that this requirement is imposed only at the finer-grained level of modes, rather than at the level of complete actors. Thus, different modes of *A* can have different dataflow rates associated with them. The requirement of constant mode-level dataflow rates and the potential for heterogeneous dataflow behavior between modes provides a useful combination of expressive power and analysis potential when applying CFDF [e.g., see Plishker et al. (2009)].

### 3.6. Architecture design

**Figure 1:**
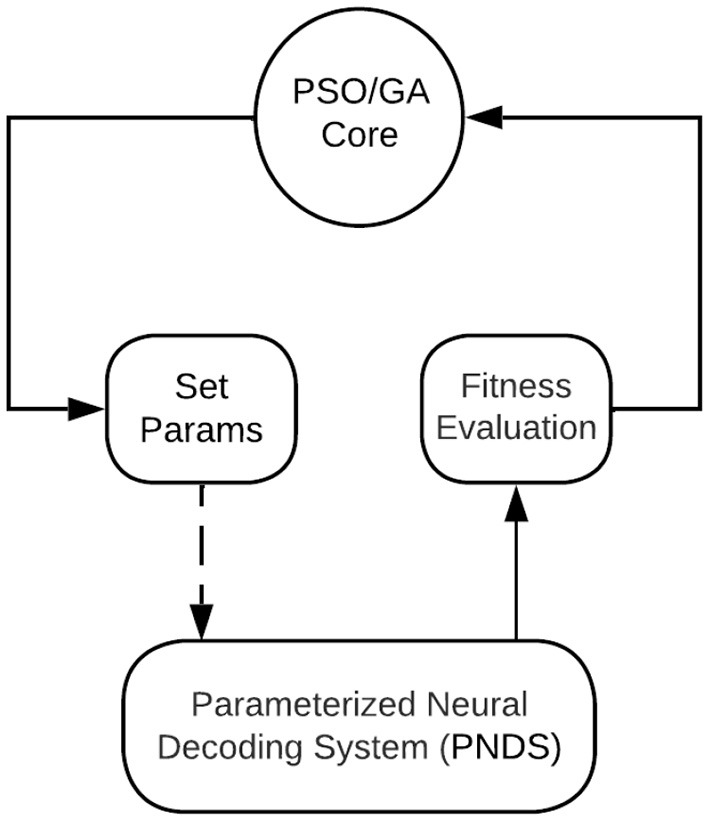
An illustration of the overall architecture of the NEDECO platform. The PSO/GA Core means the actor could be either PSO Core actor or GA Core actor.

Figure 1 illustrates the overall architecture of the NEDECO platform. The platform is developed using the lightweight dataflow (LIDE) package, which is a software tool that facilitates design and implementation of dataflow-based software systems and tools for signal and information processing (Lin et al., 2017). Dataflow modeling in LIDE is “lightweight” in the sense that it involves a compact set of application programming interfaces (APIs) that can easily be retargeted to different implementation languages. This makes it relatively easy to adapt LIDE for different design processes and apply it to different systems and tools, such as NEDECO.

The block in Figure 1 labeled PNDS encapsulates the neural decoding system that is being applied to NEDECO so that optimized parameter configurations can be derived. Here, PNDS stands for Parameterized Neural Decoding System. Integrating a PNDS into NEDECO involves writing a compact software layer (“wrapper”) for the PNDS so the overall decoding system can be executed as a CFDF actor within NEDECO, and so the parameters of the PNDS can be modified in a standard way, using the actor-parameter configuration mechanisms of the underlying LIDE tool. Here, we use a minor abuse of terminology where we use “PNDS” to describe both the neural decoding system whose parameters are being optimized and the actor in NEDECO, as shown in Figure 1, that interfaces the neural decoding system to overall PSO-based or GA-based optimization process. A PNDS can be plugged into the NEDECO framework either based on an existing neural decoding algorithm or based on a newly-developed algorithm.

Each firing of the PNDS actor involves executing the given neural decoding system repeatedly on a pre-defined dataset of calcium imaging based neural images, and aggregating the resulting measurements on execution time and accuracy. The wrapper functionality is responsible for iterating across the given dataset, and producing the results, encapsulated in the form of dataflow tokens, so that they can be interpreted by the enclosing PSO or GA process. Each token produced by the PNDS actor encapsulates a pair of floating-point values — one that represents the accuracy and the other that represents the execution time — where both values are averaged across measurements taken for each image in the dataset.

In Section 4, we demonstrate and experiment with NEDECO using two alternative neural decoding systems as the PNDS — NDSEP and CellSort. However, NEDECO is not limited to NDSEP and CellSort; the process of defining wrappers for integration with NEDECO can be applied flexibly to other neural decoding systems, which significantly broadens the applicability of the platform.

The PNDS itself need not be implemented using CFDF nor any other kind of dataflow methods; only the CFDF-based wrapper is needed to ensure its proper integration into NEDECO. Between the two PNDSs that we experiment with in Section 4, NDSEP is implemented using LIDE and its associated CFDF support, while CellSort is implemented independently of LIDE, and without any explicit connection to dataflow modeling.

The PSO/GA Core block in Figure 1 executes the core of the PSO optimization process. This is a PSO or a GA implementation that is encapsulated as a CFDF-based dataflow actor so that it can be connected with arbitrary PNDSs that have associated NEDECO wrappers. More details on the PSO Core actor and GA Core actor are discussed in Section 3.7, and Section 3.8, respectively. The Set Params block in Figure 1 receives tokens that encapsulate PNDS parameters and associated values that should be used to change those parameters in the next execution of the PNDS. A dashed edge is drawn in Figure 1 to connect the Set Params block to the PNDS because this is not a dataflow connection. Instead, this is a connection that involves calling interface functions associated with the PNDS wrapper to set parameter values in the PNDS.

The Set Params block is not a pure dataflow actor since it communicates with another actor using mechanisms other than the passage of tokens through edges. The general approach of integrating non-dataflow parameter manipulation with parameterized dataflow subsystems is useful in many contexts of signal processing system design (Bhattacharyya et al., 2019).

The Set Params block manages parameters in a general way so it just needs to be reconfigured (rather than re-implemented) when a new PNDS is applied to NEDECO. Reconfiguring the Set Params block involves providing a set of pointers to parameter values in the PNDS that can be varied by the PSO or GA, along with the sizes of the data types associated with the parameters.

The Fitness Evaluation block in Figure 1 is a simple actor that applies the linear aggregation function discussed in Section 3.3 to the results produced by the PNDS actor. The actor can easily be replaced by alternative actors (or extended in a parameterized way within the same actor) to perform different aggregation functions. We anticipate that future developments in NEDECO will include incorporation of alternative aggregation functions to facilitate experimentation with this aspect of the PSO-based and GA-based approach.

Further workflow details for the overall NEDECO architecture are presented in the Supplementary material (Algorithm S1).

To summarize the flow of data in the optimization loop represented in Figure 1, we start by observing that each token produced by the PSO Core actor or the GA Core actor corresponds to a single PSO particle or a single GA chromosome, which in turn corresponds to a single candidate solution (parameter configuration) for the PNDS. Each token produced by the PNDS actor provides evaluation results — accuracy and execution time — for a given candidate solution. Each firing of the Fitness Evaluation actor simply converts the accuracy and execution results into a single floating point value through a linear aggregation of the two components of the input token. The update-population mode of the PSO Core actor and the update-crossover and update-complete modes of the GA Core actor, which are the most important modes of the core actors, each consume a block of *P* fitness evaluation results (for all candidate solutions in the current population), and use these results to generate the next generation of the population. The resulting new candidate solutions are output, as a block of *P* tokens upon completion of each of the update-population, update-crossover, and update-complete modes.

### 3.7. PSO Core actor

In this section, we present design details for the PSO Core actor, and in the following section we provide an overview of the GA Core actor. These two actors are key components of NEDECO, and are also envisioned to be applicable to a wide variety of other parameter optimization contexts in computational neuroscience. The PSO and GA optimization algorithms share similar operating processes, including population initialization, iterative mutation of individuals within the population, and iterative fitness evaluation.

The PSO Core actor has six CFDF modes, which are referred to as the initialize-write, initialize-read, update-population-write, update-population-read, stopping-evaluation, and write-output modes.

**Figure 2:**
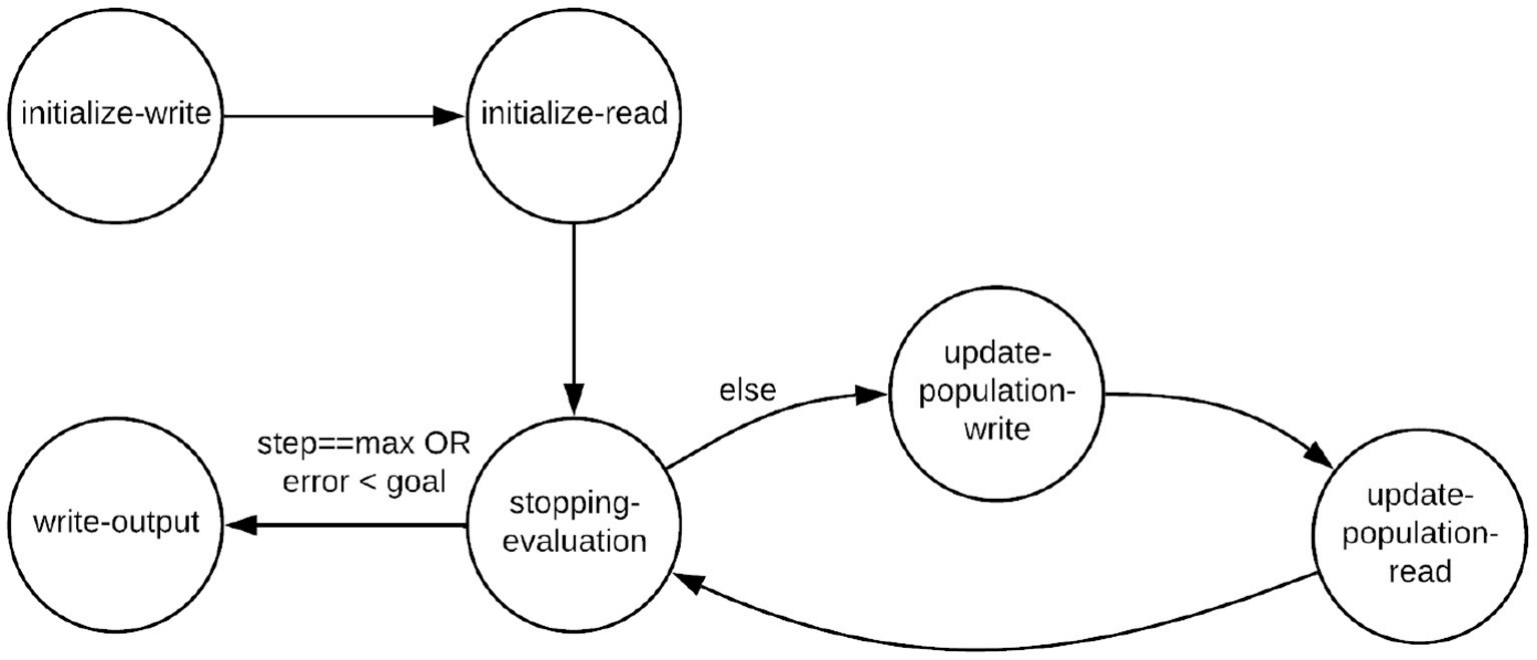
Mode transition graph for the PSO Core actor.

Figure 2 illustrates the *mode transition diagram*, which is a graphical representation for a CFDF actor that shows how the current mode of the actor transitions from one firing to the next. A mode transition diagram is a general method for characterizing CFDF actors. As part of the general design rules for CFDF actors, the mode that a given firing transitions to is determined as part of the functionality that implements the mode (Plishker et al., 2009).

Table 1 represents the dataflow table for the PSO Core actor. Like the mode transition diagram, the dataflow table is another general way for characterizing a CFDF actor. The rows of this table correspond to different modes of the actor and the columns correspond to different ports (incidences with input and output edges). For each output port of a CFDF actor, the corresponding column in the actor's dataflow table specifies the number of tokens produced in each mode, and similarly, for each input port, the corresponding column gives the *negative of* the number of tokens consumed from the port in each mode.

**Table 1:** Dataflow table for the PSO Core actor.

**Mode**	**Input**	**Output**
Initialize-write	0	*P*
Initialize-read	-*P*	0
Stopping-evaluation	0	0
Update-population-write	0	*P*
Update-population-read	-*P*	0
Write-output	0	0

For the PSO Core actor, the parameter *P* gives the number of particles in the PSO population. Thus, for example, we see from the table that in the update-population-write mode, the actor produces *P* tokens onto the actor's output edge. In the update-population-read mode, the actor consumes the same number of tokens from the input edge.

In a given execution of NEDECO, the PSO actor starts in the initialize-write mode. In this mode, the actor reads PSO parameters, such as the population size *P* and the number and types of PNDS parameters. The initialize-write mode then constructs the initial swarm population by randomly generating *P* particles. After generating the initial PSO population, the initialize-write mode outputs *P* tokens on the actor's output edge, where each token encapsulates a pointer to a distinct particle in the initial population. Then the actor mode is transitioned to initialize-read.

In the initialize-read mode, the PSO Core actor consumes a block of *P* fitness evaluation results, which correspond to computed fitness values for the different configurations of the PNDS, as defined by the different particles in the current PSO population. Each of the fitness values is consumed as a single token from the input edge to update-population. These fitness values are used to initialize the best fitness value of each particles, as well as the global best solution. The mode is then transitioned to stopping-evaluation.

The update-population-write mode updates the particle velocities and positions according to the best particle fitness and positions (based on the best fitness achieved so far for a given particle) and global best fitness and positions. The PSO Core actor then produces the particles in the current population onto the actor's output edge (in a manner similar to the initialize-write mode). The actor transitions to the update-population-read mode afterwards.

In the update-population-read mode, the PSO Core actor consumes a block of *P* fitness evaluation results, similar to the initialize-read mode. The particles' best positions, best fitness and global best solutions are updated according to these fitness results. After that, the actor transitions to the stopping-evaluation mode.

In the stopping-evaluation mode, the PSO Core actor first checks whether the stopping criterion for PSO evolution has been reached. Stopping criteria can be configured by the user, and can include factors such as a maximum number of PSO iterations (generated PSO populations), and the maximum acceptable error, which defines a level of accuracy that, when reached, triggers termination of the PSO even if the maximum number iterations has not yet been reached. Details on the stopping criteria used in our experiments are discussed in Section 4. If the stopping criterion for the PSO Core actor is reached, then the actor transitions to the write-output mode. Otherwise, transitions back to update-population-write mode.

The write-output mode simply writes the final results of the PSO-based optimization process to a set of output files. The generated output includes the values of the optimized parameter settings — as determined by the best particle within the final PSO population — as well as diagnostic output that can be used to gain insight into how the optimization process evolved through different generations of the population. If the best particle is not unique — that is, if multiple parameter settings achieve the maximum fitness value — then the the parameter settings associated with all of the tied-for-best particles are written in the output.

The overall time complexity per iteration of NEDECO using PSO optimization is *P* × *F* where *F* denotes the fitness evaluation time complexity when PNDS runs one time on the training dataset. No complexity expression for *F* is provided in PNDS, which are NDSEP (Lee et al., 2020) and CellSort (Mukamel et al., 2009) in our experiments, so it's difficult to derive because of third party functions involved.

A pseudocode sketch of NEDECO integrated with the PSO Core actor is given in Algorithm 1.

**Algorithm 1:** A pseudocode sketch of NEDECO integrated with the PSO Core actor.

/^*^ PSO actor initialize-write mode ^*^/
for each particle **do**
Initialize particle position and velocity
Evaluate fitness function
Send particle parameters to evaluate
end **for**
PNDS actor fired to evaluate parameters
/^*^ PSO actor initialize-read mode ^*^/
for each particle **do**
Read fitness values
Initialize Pbest, Gbest.
end **for**
while Not reach max iterations or error criteria **do**
/^*^ PSO actor stopping-evaluation mode ^*^/
/^*^ PSO actor update-population-write mode ^*^/
for each particle **do**
Update particle velocity and position
Send particle parameters to evaluate
end **for**
PNDS actor fired to evaluate parameters
/^*^ PSO actor update-population-read mode ^*^/
for each particle **do**
read fitness values
Update Pbest
end **for**
update Gbest if necessary
end **while**
/^*^ PSO actor write-output mode ^*^/
write results to output files

### 3.8. GA Core actor

**Figure 3:**
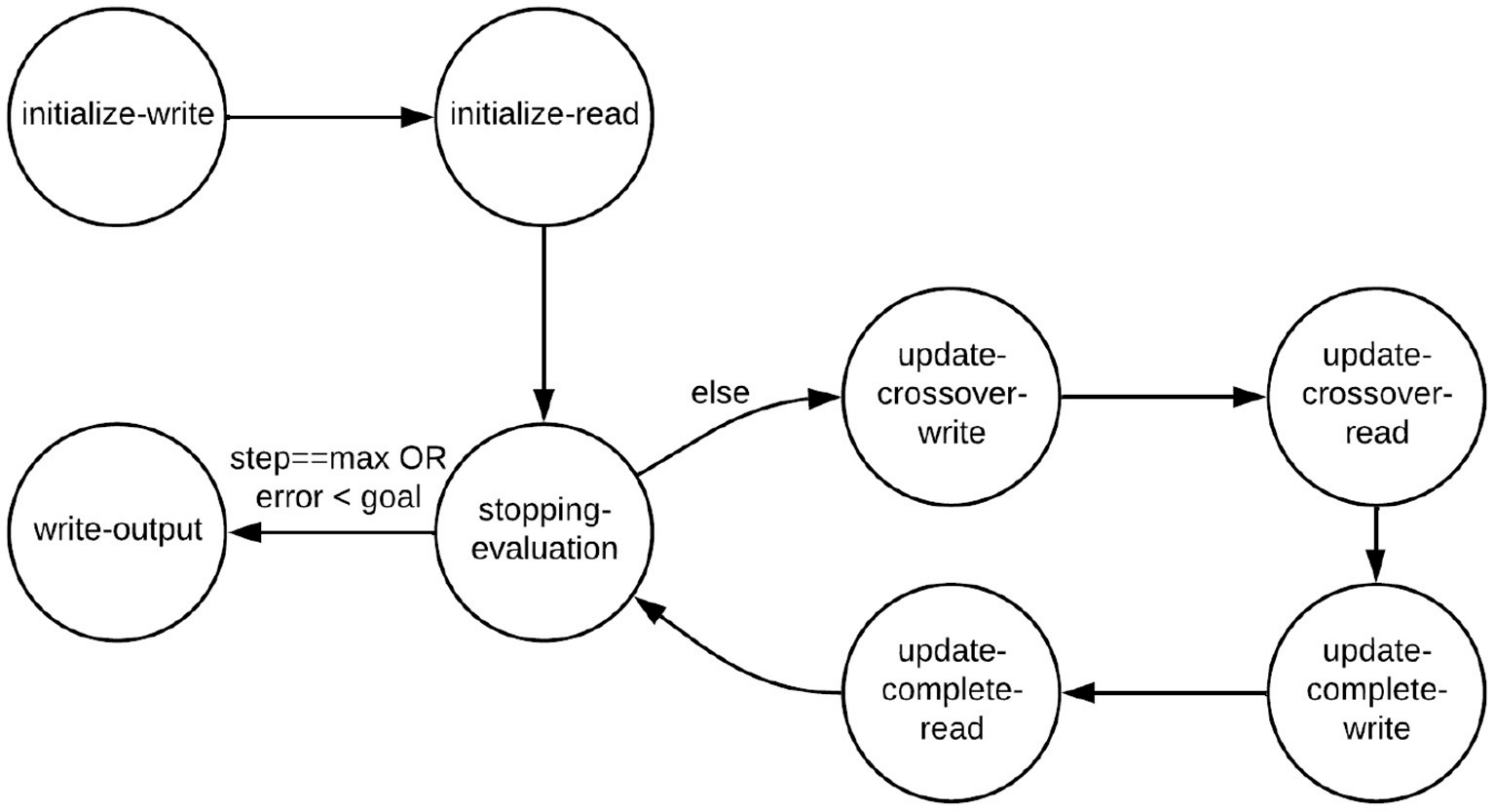
Mode transition graph for the GA Core actor.

To use a GA as the search strategy instead of a PSO, we simply replace the PSO Core actor in NEDECO with the GA Core actor. The GA Core actor has eight modes, as illustrated in Table 2. This table shows the dataflow table of the actor. Here, *P* denotes the number of individuals in the GA population, and *CP* is the number of individuals that are selected for crossover in each GA iteration. The GA implementation in NEDECO supports elitism (Back et al., 1997) and has an associated non-negative integer parameter *EL*. If *EL*>0, then the top *EL* individuals, based on fitness evaluation, are unconditionally carried over from one generation to the next. The mode transition graph is illustrated in Figure 3. The GA Core actor is designed in a manner similar to the PSO actor; further details on the GA Core actor design are omitted from the paper for brevity; details can be found in the Supplementary material (Algorithm S2).

**Table 2:** Dataflow table for the GA Core actor.

**Mode**	**Input**	**Output**
Initialize-write	0	*P*
Initialize-read	-*P*	0
Stopping-evaluation	0	0
Update-population-crossover-write	0	*CP*-*EL*
Update-population-crossover-read	-(*CP*-*EL*)	0
Update-population-complete-write	0	*P*-*CP*
Update-population-complete-read	-(*P*-*CP*)	0
Write-output	0	0

The time complexity per iteration of NEDECO using GA optimization is (*P*−*EL*) × *F*, where *F* denotes the fitness evaluation time complexity when PNDS operates once across the entire training dataset. The setting of *EL* that we use in our experiments is included in Table 3.

**Table 3:** PSO and GA configuration settings.

	**Hyper-parameter**	**Value**
PSO	Number of particles	24
	Neighborhood strategy	Ring
	Neighborhood size	10
	*c*_1_	1.496
	*c*_2_	1.496
	*w*_*min*_	0.3
	*w*_*max*_	0.7298
GA	Population size	24
	Selection method	Roulette wheel selection
	Crossover method	One-point crossover
	Mutation method	Single point mutation
	Crossover rate	0.5
	Mutation rate	0.05
	Selective pressure	1.5
	Elite population size	1
	Mating population size	24

Our implementation of the GA Core actor utilizes modified versions of code from Mallet (2022).

### 3.9. Accelerated execution of the PNDS actor

Parallel computing is commonly applied to PSO implementations to help reduce the time required for optimization. In NEDECO, the execution time is vastly dominated by that of the neural decoding system that is being optimized. Recall that the neural decoding system must be executed across the given dataset for every particle in every population generation until the PSO terminates. However, execution of multiple particles in the same population can be conducted in parallel if adequate computing resource are available. The dataflow-based model of NEDECO is utilized for efficient and reliable exploitation of parallelism for particle evaluation when NEDECO is operated on a multi-core computing platform.

For efficient multi-threaded execution on a multi-core platform, each firing of the PNDS actor is encapsulated within a separate thread, and blocks of *N*_*t*_ firings of the PNDS actor are executed concurrently, where *N*_*t*_ is a user-defined parameter number that specifies the number of threads allocated to NEDECO. The default setting of *N*_*t*_ is simply *N*_*t*_ = *N*_*c*_, where *N*_*c*_ is the number of cores or virtual cores (if hyper-threading is present) on the targeted processing platform. In Section 4, we include experimental results on the measured execution time improvement of NEDECO that is achieved through the incorporated features for accelerating particle evaluation.

When applying this multi-threaded approach to PNDS acceleration, it should be noted that the measured execution time for each particle evaluation is the single-threaded performance of the associated neural decoding system configuration. If the optimized neural decoding system will be deployed as a multi-threaded implementation, then the single-threaded performance evaluated within NEDECO can be viewed as an estimate to help compare the relative processing complexity of alternative PNDS configurations. An interesting direction for extending NEDECO is to incorporate the ability to allocate multiple threads to the evaluation of individual particles, which can provide a better estimate of performance for a neural decoding system that will ultimately be deployed in multi-threaded form.

A useful feature of the existing acceleration approach in NEDECO is that it can be applied uniformly to any neural decoding system that NEDECO is applied to — that is, regardless of whether or not a multi-threaded implementation is available for the system.

## 4. Experiments

In this section, we demonstrate the utility of NEDECO through experiments using two different calcium-imaging based neural decoding systems. The systems that we apply in our experiments as alternative PNDSs are the Neuron Detection and Signal Extraction Platform (NDSEP) (Lee et al., 2020), and CellSort (Mukamel et al., 2009). NDSEP is implemented in C++ so its integration with NEDECO, which is also implemented in C++, requires no need for interfacing between different programming languages. On the other hand, CellSort is developed in MATLAB. The integration of CellSort with NEDECO therefore helps to demonstrate the flexibility of NEDECO in being usable with neural decoding systems that are developed in languages other than the native language of NEDECO. To integrate CellSort with NEDECO, features available in MATLAB for interfacing with C++ code are utilized in the CFDF wrapper that is used to interface CellSort to the rest of NEDECO.

When integrating a specific neural decoding system X into NEDECO, we refer to the resulting setup for optimizing the configuration of X as *NEDECO-X*. Thus, our experiments in this section include experiments with NEDECO-NDSEP and NEDECO-CellSort.

By default NEDECO uses PSO as its underlying optimization strategy; however, NEDECO can be readily adapted to work with other population-based optimization strategies. If NEDECO is adapted to use a specific optimization strategy Y and integrated with a specific neural decoding system X, we refer to the resulting configuration as NEDECO-Y-X. If Y is not specified, then it is assumed to be the default strategy PSO. In addition to experiments with NEDECO-NDSEP and NEDECO-CellSort, this section also includes experiments with NEDECO-GA-CellSort an NEDECO-GA-NDSEP, where GA stands for “genetic algorithm.”

Experiments are repeated 10 times on each training/testing group with the same configuration. Each of the 10 repetitions for a given group is based on a different seed for the random number generator used by NEDECO, which in turn generally leads to a different initial population for the PSO.

All of the experiments reported on in this section were performed using a 6-core (Intel i7-8700 @3.2GHz) desktop platform that supports up to two simultaneously-executing threads per core. The platform is equipped with 16 GB RAM.

### 4.1. Fitness function

The fitness function of a PSO measures the quality of a given candidate solution with respect to the optimization objective of the PSO. As described in Section 3.6, the fitness function is implemented in the Fitness Evaluation block of Figure 1, and the function is configured as a linear aggregation of measurements on neuron detection accuracy and execution time.

In our experiments, the accuracy component of the fitness function is configured in terms of the Overall Dice Coefficient (ODC) for neuron detection, which intuitively captures the ratio of the overlap between detected and ground truth neurons to the area of the neuron mask, where the area is measured in terms of number of pixels. By definition, ODC values always lie within the interval [0, 1]. For details and motivation on using the Dice coefficient to measure accuracy for image segmentation problems, we refer the reader to Zou et al. (2004). For a given calcium imaging dataset, we typically have a single neuron mask, and the neuron detection process produces a single overall detection result for the dataset. Thus, the ODC provides a measure of neuron detection accuracy for an entire dataset in the context of NEDECO. It is for this reason that we use “overall” in the term ODC. When computing the ODC, if there are more than one detections that match a single neuron in the ground truth, then only the best match having the highest overlapping portion is kept. On the other hand, if a detection matches multiple neurons in ground truth, only the best match is taken into consideration.

The execution time component τ(*p*) of the fitness function of each particle *p* is normalized to be in the interval [0, 1], which is the same interval that defines the range of ODC. In particular, τ(*p*) is defined by $\tau \left(p\right)=\frac{W\left(p\right)}{W_{max}\left(p\right)}$, where *W*(*p*) is the execution time measured for the most recent evaluation of *p* (using the PNDS actor), and *W*_*max*_ is the maximum execution time observed so far, across all particles in all populations that have been generated and evaluated through the current PSO iteration.

The fitness function is a weighted harmonic mean of the accuracy and execution time assessments, as defined above; for background on fitness functions of this form, we refer the reader to Reyes-Sierra et al. (2006). The fitness function *F*(*p*) value for a given particle *p* in NEDECO can be expressed as:


(1) $$\begin{array}{l} F\left(p\right)=1-\frac{1}{\frac{a_{1}}{D\left(p\right)}+\frac{a_{2}}{TimeScale}}; \end{array}$$

(2) $$\begin{array}{l} TimeScale=1-\tau \left(p\right); \end{array}$$

(3) $$\begin{array}{l} a_{1}+a_{2}=1. \end{array}$$

Here, *a*_1_ and *a*_2_ are both positive-valued weights for the component objectives, as discussed in Section 3.3. A higher value of *a*_1_ corresponds to a higher relative importance weighting for accuracy compared to execution time. Unless stated otherwise, we use the setting *a*_1_ = 0.8, *a*_2_ = 0.2, which is representative of cases in which accuracy is the emphasized objective but some consideration to execution time is also important.

### 4.2. Dataset

In our experiments to evaluate NEDECO-NDSEP and NEDECO-CellSort, we employ the Anterior Lateral Motor Cortex (ALM) dataset (Li et al., 2015). ALM is a real-world two-photon calcium imaging dataset that is acquired from the anterior motor cortex in mice while the mice perform a tactile delay-response task. The dataset includes 11 189 frames of calcium imaging data. As a pre-processing step, we apply CaImAn-NoRMCorre (Giovannucci et al., 2019) for motion correction. The objective of this preprocessing step is to eliminate the influence of motions on the model training and evaluation.

To evaluate NEDECO on the ALM dataset, we employ *k*-fold cross-validation with *k* = 3. The 11 189 calcium imaging frames are randomly split into 3 folds. In each of the three experiment groups, corresponding to our selection of *k* = 3, we select a different fold to be the testing dataset, while the other two datasets are used for training. Here, by “training,” we mean executing NEDECO to optimize parameters, while by “testing,” we mean evaluating the optimized system configuration that results from the training process. The temporal order of the frames in the training and testing sets is retained to keep temporal features intact.

### 4.3. PSO and GA actor configuration

Table 3 lists PSO configuration settings and GA settings that are used in our experiments with both NDSEP and CellSort. These settings were derived empirically by experimenting with the PSO and GA under different configurations. Background on the first two PSO parameters listed in Table 3 is included in Section 3. Discussion of the other parameters in the table is beyond the scope of this paper; for background on these parameters, we refer the reader to Clerc and Kennedy (2002) and Hopgood and Mierzejewska (2008). The stopping criterion (see Section 3.7) for both the PSO and GA is set to 50 iterations in all experiments.

### 4.4. NDSEP

We first demonstrate NEDECO by using it to optimize parameters of NDSEP, which is designed to be a real-time neural decoding system for calcium imaging. NDSEP takes as input a video stream captured by a miniature calcium imaging device as input, and produces as output a set of detected neuron masks as well as extracted neuron signals corresponding to the masks. Details of NDSEP for neuron detection are in Lee et al. (2020).

Table 4 lists parameters in NDSEP that need to be configured. For details on the meanings of these parameters, we refer the reader to Lee et al. (2020). The range of each parameter is given in Table 4 along with an indication of whether the parameter is continuous-valued or integer-valued. The admissible ranges of the parameters are imposed based on limits on the parameter values that defined by NDSEP. However, even with these limitations imposed on the parameter value ranges, the overall search space of parameter combinations is extremely large and prohibitively expensive for exhaustive evaluation (theoretically, the search space is infinite since some parameters are continuous-valued).

**Table 4:** Parameters in NDSEP.

**Parameter**	**Type**	**Minimum value**	**Maximum value**
Threshold step	Continuous	10.0	100.0
Minimum circularity	Continuous	0.0	1.0
Minimum convexity	Continuous	0.0	1.0
Minimum inertia ratio	Continuous	0.0	1.0
Gaussian blur size	Integer	1	60
Gaussian standard	Continuous	0.0	1.0
Median blur size	Integer	1	60

Table 5 shows training and testing results for NEDECO-PSO-NDSEP and NEDECO-GA-NDSEP using the aforementioned *k*-fold cross-validation approach, where *k* = 3. Experiments are repeated 10 times on each fold. The average ODC and frame rate values resulting from the training and testing datasets are shown in Table 5, where the average and standard deviation are taken over all 30 experimental runs. Here, the frame rate *ρ*, which is the average time to execute neuron detection on a single frame, is calculated as: $\rho =\frac{n_{f}}{t_{tot}}$, where *n*_*f*_ is the number of image frames in the given dataset, and *t*_*tot*_ is the total processing time for the dataset.

**Table 5:** Training and testing results for NEDECO-PSO-CellSort and NEDECO-GA-CellSort.

**Method**	**Training**				**Testing**			
	**Dice coefficient**	**Recall**	**Precision**	**Frame rate (fps)**	**Dice coefficient**	**Recall**	**Precision**	**Frame rate (fps)**
PSO	0.5968	0.6853	0.9380	207.9	0.5549	0.6292	0.9167	206.9
	(±0.0069)	(±0.0185)	(±0.0429)	(±23.5000)	(±0.0120)	(±0.0298)	(±0.0315)	(±23.8284)
GA	0.5781	0.6908	0.8894	186.4498	0.5310	0.6329	0.8904	186.8505
	(±0.0157)	(±0.0798)	(±0.0188)	(±44.2536)	(±0.0105)	(±0.0443)	(±0.0577)	(±23.7246)

Table 5 also shows the average and standard deviation values for recall and precision results among the 30 repeated experiments. Here, precision and recall are calculated for the neuron detection results. A threshold *T*_*dice*_ is used to distinguish between true and false positives in neuron detection results — if the Dice coefficient λ(*δ*) for a given detection *δ* is greater than or equal to *T*_*dice*_, then the detection is categorized as a true positive (TP) result; on the other hand, if λ(*δ*) < *T*_*dice*_, then the detection is categorized as a false positive (FP) result. For the precision and recall results in Table 5, we use *T*_*dice*_ = 0.5.

**Figure 4:**
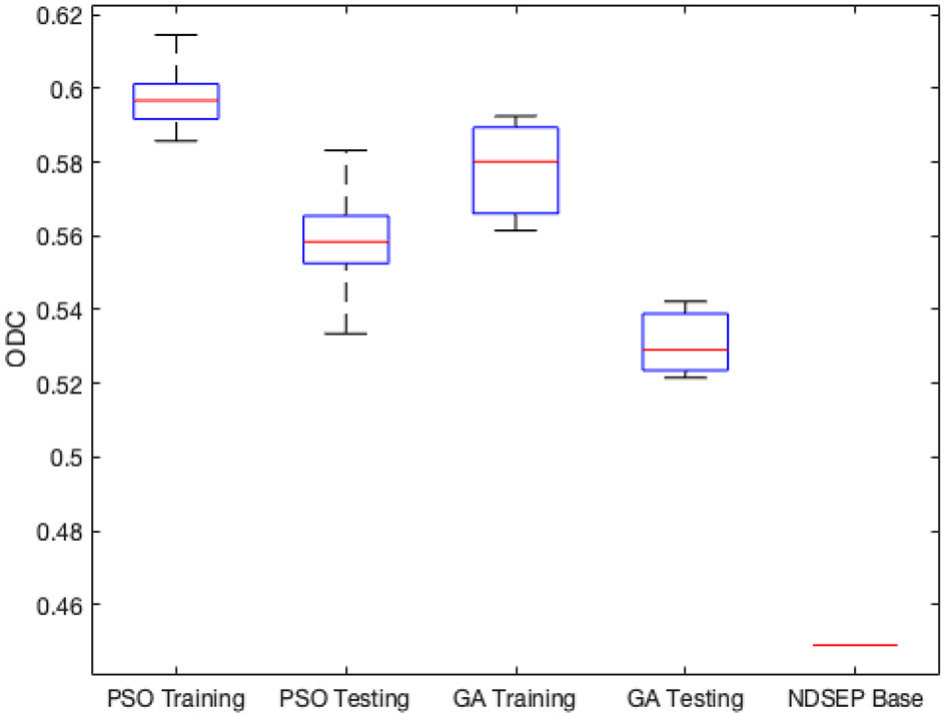
Minimum, maximum, quartiles, and average of the ODC over all *k* = 3 experimental groups for NEDECO-NDSEP.

Figure 4 shows the minimum, maximum, quartiles, and average of the ODC over all 30 experimental runs (across all 3 groups) for the training and testing datasets. The right side of Figure 4 also shows the ODC achieved by the *base NDSEP configuration*, which is the original hand-optimized configuration of NDSEP (Lee et al., 2020).

Compared with the hand-optimized Base NDSEP configuration, NEDECO-PSO-NDSEP and NEDECO-GA-NDSEP demonstrate major advantages. In Figure 4, we see that both NEDECO-PSO-NDSEP and NEDECO-GA-NDSEP consistently achieve significantly higher ODC values compared to Base NDSEP. The recall and precision levels achieved by Base NDSEP are 0.725 and 0.495, respectively. From Table 5, we see that the recall levels produced by NEDECO-NDSEP are marginally worse compared to Base NDSEP, however, the precision produced by NEDECO-NDSEP is much better. The slightly improved recall of NDSEP results because missed detections are intuitively easier to notice, whereas multiple detections on the same ground truth neuron are more difficult avoid, especially when the detections correspond to very small regions of interest. Moreover, the frame rates shown in Table 5 are also significantly higher compared to the frame rate provided by Base NDSEP, which is 149.9 fps. This demonstrates the potential for increased execution time performance provided by NEDECO.

**Figure 5:**
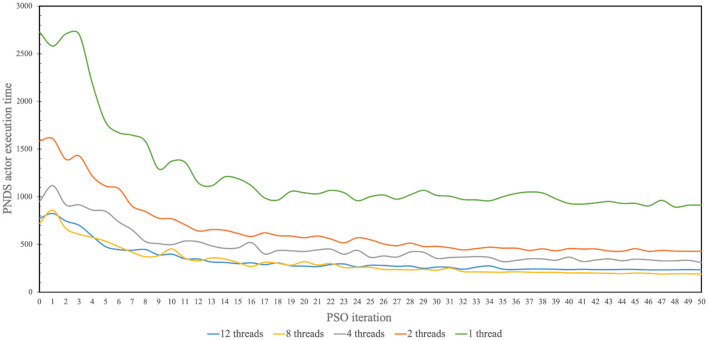
Measured results produced from use of multi-threading to accelerate operation of NEDECO-NDSEP.

Figure 5 illustrates measured results from the application of multi-threading to accelerate firings of the PNDS actor in NEDECO-NDSEP. Recall that a firing of the PNDS actor corresponds to execution of the neural decoding system (NDSEP in this case) on the entire given dataset. The horizontal axis in the figure corresponds to successive PSO iterations; recall that in each PSO iteration, the PNDS actor fires once to evaluate all of the particles in the current population. The vertical axis corresponds to the measured execution time for firings of the PNDS actor. The different curves in the figure correspond to different numbers of threads (i.e., different values of *N*_*t*_). For each setting of *N*_*t*_ shown in Figure 5, we executed NEDECO-NDSEP 10 times, and averaged the results at each PSO iteration number to derive curve associated with *N*_*t*_.

From the results shown in Figure 5, we see significant improvements in execution time with increasing use of threads with the improvements saturating for each value of *N*_*t*_ for later PSO iterations. The only exception in terms of increasing values of *N*_*t*_ is for *N*_*t*_ = 12, which performs marginally worse than *N*_*t*_ = 8. It is anticipated that this trend for higher values of *N*_*t*_ is due to factors such as excessive contention for resources among the allocated threads or overheating in the processor.

**Figure 6:**
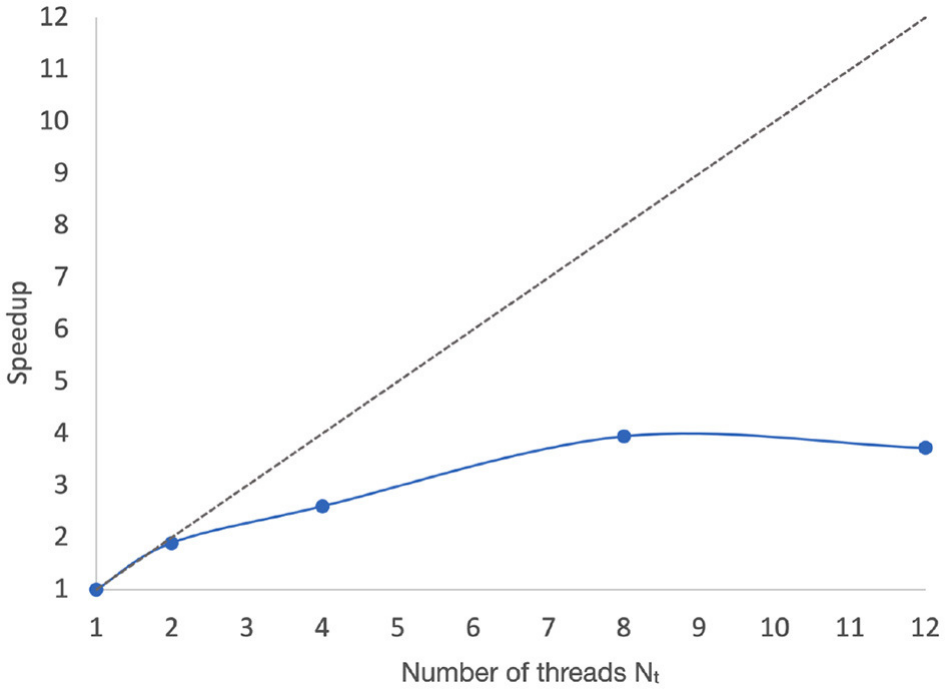
Speedups associated with the data illustrated in Figure 5.

Figure 6 shows the measured speedups associated with the data illustrated in Figure 5. Here, the speedup associated with *N*_*t*_ = *p* is calculated as $\frac{T_{1}}{T_{p}}$, where *T*_1_ and *T*_*p*_ are corresponding execution time measurements associated with single-threaded (sequential) execution and execution with *N*_*t*_ = *p*. *T*_1_ and *T*_*p*_ are calculated by averaging the PNDS actor execution time over 50 iterations, as shown in Figure 5.

These execution time improvements reflect not only faster operation of the NEDECO optimization process, but also improvements in the average frame rate associated with PSO particles (candidate NDSEP configurations).

### 4.5. CellSort

CellSort is a widely used Matlab-based tool for neuron instance segmentation and signal analysis (Mukamel et al., 2009). The CellSort algorithm for deriving neuron segmentation masks involves three main steps — Principle Component Analysis (PCA), Independent Component Analysis (ICA), and Image Segmentation. Like NDSEP, CellSort involves several parameters that need to be set by the user. These parameters have a major impact on the segmentation results.

Moreover, CellSort runs much more slowly compared to NDSEP, which leads to a corresponding slowdown for the PSO-based optimization process of NEDECO-CellSort. However, from our experiments, we found that a large portion of the time in CellSort is spent in the PCA step, which has no associated parameters that need to be configured by the user. Thus, the PCA step can be executed as a pre-processing step to the PSO optimization process, and there is no need to include PCA computation as part of the PNDS actor.

Table 6 summarizes the parameters involved in configuring CellSort. These parameters are jointly optimized by NEDECO-PSO-CellSort and NEDECO-GA-CellSort. The parameters PCl, PCf, and mu are associated with the ICA step of CellSort. These parameters respectively specify the first principal component to be kept for dimension reduction, the last principal component to be kept, and the weight to use for temporal information in the spatial-temporal ICA process. The other four parameters in Table 6 are associated with the image segmentation step. The smwidth parameter gives the standard deviation for the Gaussian smoothing kernel; the areal and areah parameters respectively give the minimum and maximum areas (in pixels) for segments that are to be retained; and the thresh parameter gives the threshold for spatial filters.

**Table 6:** Parameters in CellSort.

**Parameter**	**Type**	**Minimum value**	**Maximum value**
PCl	Integer	1	60
PCf	Integer	61	150
mu	Continuous	0.0	1.0
smwidth	Continuous	0.0	10.0
areal	Integer	50	500
areah	Integer	501	2000
Thresh	Continuous	1.5	10.0

**Figure 7:**
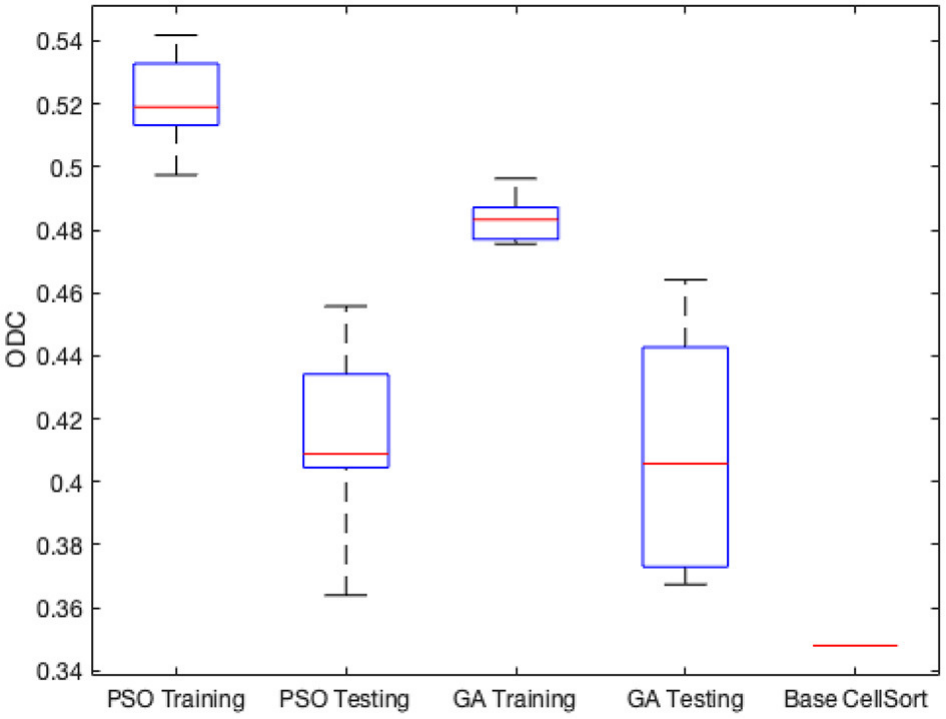
Minimum, maximum, quartiles and average of the ODC over all *k* = 3 experimental groups for NEDECO-CellSort.

We used the ALM dataset to demonstrate and experiment with NEDECO-CellSort. For NEDECO-CellSort, we used the same 3-fold cross-validation approach that is described in Section 4.2, and we averaged across 10 trials for each experiment setting. We ran experiments using NEDECO-CellSort that parallel the experiments for NEDECO-NDSEP reported in Table 5 and Figure 4. The corresponding results for NEDECO-CellSort are shown in Table 7 and Figure 7, respectively. To measure precision and recall, we used the same threshold setting, *T*_*dice*_ = 0.5, that we used for NEDECO-NDSEP (see Section 4.4). In Table 6, we list the execution time (over the whole dataset) instead of the frame rate because CellSort is not designed to operate in a real-time fashion, with frame-by-frame input/output.

**Table 7:** Training and testing results for NEDECO-PSO-CellSort and NEDECO-GA-CellSort.

**Method**	**Training**				**Testing**			
	**Dice coefficient**	**Recall**	**Precision**	**Time(sec)**	**Dice coefficient**	**Recall**	**Precision**	**Time(sec)**
PSO	0.5169	0.5352	0.8112	20.4834	0.4138	0.3992	0.7425	21.38
	(±0.0146)	(±0.0365)	(±0.0683)	(±10.0690)	(±0.0253)	(±0.0410)	(±0.0739)	(±13.5110)
GA	0.4833	0.4824	0.7784	40.9305	0.4106	0.3623	0.8259	25.9015
	(±0.0074)	(±0.0527)	(±0.1094)	(±10.1727)	(±0.0384)	(±0.0586)	(±0.1011)	(±7.7650)

The Base CellSort configuration (see Figure 7) used in our experiments was derived through a labor-intensive hand-optimization process using the ALM dataset. This optimization process took approximately three weeks. The parameter settings resulting from this hand-optimization process are discussed in Lee et al. (2020). NEDECO-CellSort eliminates the need for such labor-intensive hand-optimization, and at the same time, produces significantly more accurate and efficient configurations of CellSort.

From Table 7 and Figure 7, we see that trends for accuracy metrics (ODC, recall and precision) and execution time are similar for NEDECO-X-CellSort as those for NEDECO-X-NDSEP presented in Section 4.4. The recall and precision levels achieved by Base CellSort are 0.7101 and 0.3043, respectively. From Table 5, we see that the recall levels produced by NEDECO-X-CellSort are worse compared to Base CellSort, however, the precision produced by NEDECO-X-CellSort is better, and the degree of improvement in precision significantly exceeds the degree of reduction in recall. The F1 score, which can be viewed as an aggregation of the precision and recall metrics, for the NEDECO-PSO-CellSort and NEDECO-GA-CellSort results are 0.5132 and 0.5037, respectively, which represent improvements compared to the F1 score of 0.4260 for Base CellSort.

We measured the execution time of Base CellSort to be be 52.77 seconds (again averaged over 10 trials). This is significantly slower than the training- and testing-time averages of 20.48 and 21.38 seconds for NEDECO-PSO-CellSort, and 40.93 and 25.9015 seconds for NEDECO-GA-CellSort, as shown in Table 7.

In summary, our experiments demonstrate that NEDECO greatly reduces the effort involved in optimizing the parameters of CellSort while producing configurations of CellSort (for the different cross-validation groups) that are significantly more accurate in terms of F1 score and ODC, and also significantly more efficient compared to the baseline, hand-optimized version of CellSort.

### 4.6. Multiobjective optimization

In this section, to evaluate the linear aggregation multiobjective approach described in Section 3.3, we perform a comparison with NSGA-II. NSGA-II is a widely used multiobjective genetic algorithm. For details on NSGA-II, we refer readers to Deb et al. (2000). In our experiments, NEDECO-PSO-CellSort is compared with NSGA-II-CellSort, where we optimize CellSort using the MATLAB-based NSGA-II Package (Seshadri, 2022). Unlike our previous experiments that fix the values of *a*_1_ and *a*_2_ (see Section 4.1), NEDECO-PSO-CellSort is trained with different values of (*a*_1_, *a*_2_) — in particular, *a*_1_ is increased from 0.1 to 0.9, and *a*_2_ is correspondingly decreased from 0.9 to 0.1. The increase/decrease is performed with a step size of 0.1. NEDECO-PSO-CellSort is trained 9 times with the different settings for (*a*_1_, *a*_2_) to derive 9 Pareto fronts.

In this experiment, we applied the same 3-fold cross-validation approach that is described in Section 4.2. We repeated the steps above to obtain 9 Pareto fronts in total, 3 on each fold. NSGA-II-CellSort was trained for 50 iterations as well with the same population size of 24. Training was repeated 9 times with different seeds (for random number generation), 3 on each fold.

**Figure 8:**
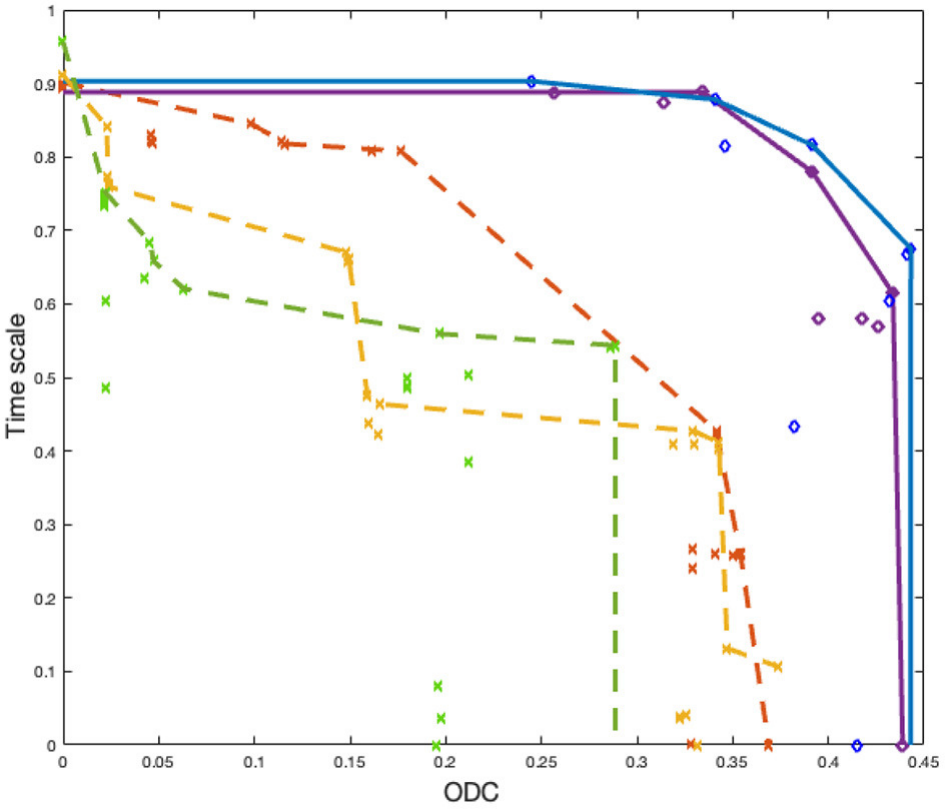
Pareto fronts derived from NEDECO-PSO-CellSort (solid lines) vs. NSGA-II-CellSort (dotted lines). Dots having the same color represent solutions derived from the same experiment. Pareto fronts are generated based on sets of dots having the same color.

Results on the test dataset are reported in Figure 8. Here, we randomly pick two Pareto fronts from NEDECO-PSO-CellSort and three from NSGA-II-CellSort. From the results, we see that NEDECO-PSO-CellSort outperforms NSGA-II-CellSort with better Pareto fronts. We use the AUC (area under the curve) (Bhowan et al., 2010) metric to evaluate the Pareto fronts. The average AUC of NEDECO-PSO-CellSort is 0.3971 while the average AUC of NSGA-II-CellSort is 0.2121. The averaging is performed across all generated Pareto fronts; in contrast, only a proper subset of Pareto fronts is shown in Figure 8 to avoid excessive clutter in the illustration. In summary, the linear aggregation multiobjective approach of NEDECO significantly outperforms NSGA-II in our experiments.

## 5. Discussion

In this paper, we have motivated the problem of parameter optimization for neural decoding systems, and we have presented a novel framework called the NEDECO package. NEDECO automatically performs parameter optimization to significantly improve the effectiveness of neural decoding systems. NEDECO applies methods from particle swarm optimization genetic algorithm, and dataflow modeling to develop a modular framework into which a wide variety of neural decoding systems can be integrated to help derive more effective configurations of the systems. NEDECO is also innovative in its consideration of multidimensional design evaluation spaces rather than being focused only on accuracy. In our current prototype of NEDECO, we have focused on the objectives of neuron segmentation accuracy and execution-time efficiency. This particular combination of design evaluation metrics is motivated by the important applications of neural decoding in real-time contexts, especially in the context of neuromodulation systems.

We have demonstrated the adaptability of NEDECO to different optimization algorithms and systems by presenting case studies of its integration with two state-of-the-art neural decoding systems — NDSEP and CellSort. The experimental results indicate significant performance improvements for both NDSEP and CellSort when their system parameters are optimized using NEDECO. Moreover, it is demonstrated that the dataflow-based design of NEDECO leads to structured and efficient utilization of multi-core computing technology to accelerate the optimization process of NEDECO.

Compared to the accuracy results demonstrated in the original work on NDSEP (Lee et al., 2020), the Base NDSEP and Base CellSort algorithms, which have manually-tuned parameters, demonstrate worse results in the experiments reported on in this paper. This is because of the more strict evaluation of neuron segmentation performance that is employed in this paper. For example, in Lee et al. (2020), when comparing with the ground truth mask, if the detected region of interest overlaps with any of the neurons in the mask, a match is counted, regardless of whether or not the ground truth neuron already has a match. However, in this paper, true positives are counted based on a threshold on the percentage of overlapping pixels associated with the match. The multiple-to-one match situation is excluded as well. By making the evaluation more strict in this way, we are able to present better insight into the accuracy performance of the investigated neural decoding systems.

From Section 4, we see similar performance trends between NEDECO-PSO and NEDECO-GA when CellSort and NDSEP are applied, respectively, as the PNDS. However, we cannot conclude from our experiments that in general NEDECO-PSO and NEDECO-GA have similar effect to optimize neural decoding systems (regardless of the PNDS used). Users benefit from the flexibility to try both search strategies — PSO and GA — for a given PNDS and operational scenario and to utilize the one that performs best. A useful direction for future work is to incorporate additional search strategies to further enhance this form of flexibility.

Due to the lack of suitable calcium imaging datasets for neuron detection, the experiments in this paper are presented only on the ALM dataset. For most existing calcium imaging datasets, the absence of ground truth results prevents the evaluation of calcium imaging processing platforms. Additionally the ground truth in the ALM dataset is a mask representing the whole dataset, so when the dataset is split into training and testing subsets, some of the neuron activity can be lost, which introduces challenges to model optimization, and can make testing performance worse than expected in terms of accuracy. The smaller the subset is, the more activity is in general lost. In terms of the *k*-fold cross validation, with a greater *k* value, the test subset will be too small to keep from losing too many neuron activities. This is why we use a small *k* value of 3 in Section 4. To address this issue, an important direction for future work is development of and experimentation with datasets that have ground truth information available at the frame level (Sità et al., 2022).

We are among the first to apply automated parameter optimization to calcium-imaging-based neural decoding and provide a dataflow-based implementation, which helps to enhance the scalability, modularity, and efficiency of the framework. Theoretically, the proposed framework can handle any parameter optimization method. The current implementation includes two parameter optimization approaches, GA and PSO. We chose these approaches because they are representative of widely-used search techniques that are relevant to complex and irregular search spaces. The focus of this paper is to demonstrate the utility of automated parameter optimization for calcium imaging based neural decoding; the focus of the paper is not to advocate for the superiority of any specific search technique.

## Data availability statement

NEDECO is being made available as open source software for the research community. The software can be accessed via https://code.umd.edu/dspcad-pub/dspcadwiki/-/wikis/software/Software. Further inquiries can be directed to the corresponding authors.

## Author contributions

JX wrote the first draft of the manuscript, conducted experiments, and iteratively revised the manuscript. RC and SB contributed to the conception and design of the study, contributed to revising the first manuscript draft, and subsequent drafts. All authors contributed to the article and approved the submitted version.
