# Supplementary material for: A parameter-optimization framework for neural decoding systems
Source: Front Neuroinform. 2023 Feb 2;17:938689. doi: 10.3389/fninf.2023.938689 (PMC9932190; doi:10.3389/fninf.2023.938689)
Supplement: Supplementary file 1 [file Data_Sheet_1.pdf]

A Parameter-Optimization Framework for Neural Decoding Systems

SUPPLEMENTARY MATERIAL: PSEUDOCODE REPRESENTATIONS OF SELECTED ALGORITHMS

Algorithm S1 A pseudocode sketch of the NEDECO architecture. Here, by the Search Core actor, we mean the PSO Core or GA Core actor, depending on which search strategy is being employed.

```
while There is at least one enabled actor in the dataflow graph do
    if The Search Core actor is enabled then

        /* Fire the Search Core actor (see Algorithm 1 and Algorithm S2 for details) */

    else if The Set Params actor is enabled then

        /* Fire the Set Params actor */
        Read tokens that encapsulate new PNDS parameter values from the input FIFO of the Set Params
                actor
        Reconfigure the PNDS (wrapper) actor by updating its parameters

    else if The PNDS actor is enabled then

        /* Fire the PNDS actor */
        Execute the given neural decoding system repeatedly on a pre-defined dataset of
                calcium-imaging-based neural images
        Aggregate the resulting measurements on execution time and accuracy
        Construct a token that encapsulates the measured accuracy and execution time
        Write this token to the output FIFO of the actor

    else if The Fitness Evaluation actor is enabled then

        /* Fire the Fitness Evaluation actor */
        Read a token that encapsulates accuracy and execution time data from the input FIFO
        Apply a linear aggregation function to calculate the fitness value
        Write the calculated fitness value to the output FIFO

    end if
end while
```

Algorithm S2 A pseudocode sketch of NEDECO integrated with the GA Core actor.

```
/* GA actor / initialize-write mode */
for each chromosome in the current population do
    Initialize the chromosome
    Send parameters in chromosome to evaluate
end for

/* Fire the Set Params, PNDS, and Fitness Evaluation actors to derive the new parameter settings for the
    PNDS (see Algorithm S1 for details) */

/* GA actor / initialize-read mode */
for each chromosome do
    Read fitness values
    Initialize best result and previous best result
end for

/* Stopping-evaluation mode */
while not (max iterations reached or error criteria met) do

    /* GA actor / update-crossover-write mode */
    Apply elitism
    Select mating population
    Start crossover on mating population
    for i ← 1 to CP-EL do
        Send the parameters for the i^th individual in the mating population
    end for

    /* Fire the Set Params, PNDS, and Fitness Evaluation actors to derive the new parameter settings for
        the PNDS (see Algorithm S1 for details) */

    /* GA actor / update-crossover-read mode */
    for i ← 1 to CP-EL do
        Read fitness values
    end for
    Finish crossing-over mating population

    /* GA actor / update-complete-write mode */
    Start mutation
    for i ← 1 to P-CP do
        Select a chromosome randomly from mating population
        Send the parameters for the i^th individual in the mutation population
    end for

    /* Fire the Set Params, PNDS, and Fitness Evaluation actors to derive the new parameter settings for
        the PNDS (see Algorithm S1 for details) */

    /* GA actor / update-complete-read mode */
    for i ← 1 to P-CP do
        Read fitness values
    end for
    Finish mutation

end while

/* GA actor / write-output mode */
Write results to output file
```
